# Spatial spillovers and nonlinear effects of urban green space on population aging in China

**DOI:** 10.3389/fpubh.2026.1756430

**Published:** 2026-03-06

**Authors:** Xiaoning Zheng, Yujue Wang, Dongmei Zhang

**Affiliations:** 1 School of Economics, Minzu University of China, Beijing, China; 2 School of Management, Universiti Sains Malaysia, Minden, Malaysia

**Keywords:** aging, China, spatial Durbin model, spatial spillover effects, threshold regression model, urban green space

## Abstract

China is undergoing the world’s most rapid demographic transition, with the pressure of population aging becoming increasingly prominent. As a core component of green infrastructure, urban green spaces are gaining attention for their role in alleviating the pressures of aging. However, existing research has primarily focused on the localized benefits of urban green spaces, with insufficient exploration of their spatial effects. This study employs panel data from 300 prefecture-level cities in China spanning 2001–2023, utilizing spatial Durbin models and threshold regression models to systematically examine the spatial effects of urban green spaces on aging. It further analyzes their significance in regional collaborative governance. Findings reveal that aging exhibits distinct spatial clustering patterns. Urban green spaces not only alleviate local aging pressures by improving environmental quality, promoting physical activity, and enhancing public health, but also generate significant spatial spillover effects that positively influence aging processes in surrounding areas. Threshold analysis further indicates that the impact of urban green spaces on aging exhibits nonlinear characteristics, with their positive effects significantly intensifying once green space coverage reaches a certain critical threshold. This study emphasizes that in today’s era of frequent population mobility and deepening regional integration, examining green space policies solely from an intra-urban perspective is insufficient to address aging challenges across administrative boundaries. Recognizing and prioritizing the spatial spillover effects of urban green spaces not only facilitates the establishment of collaborative aging governance systems across cities but also provides theoretical foundations and policy insights for advancing the Healthy China 2030 strategy and achieving balanced regional green development.

## 1 Introduction

**Figure 1:**
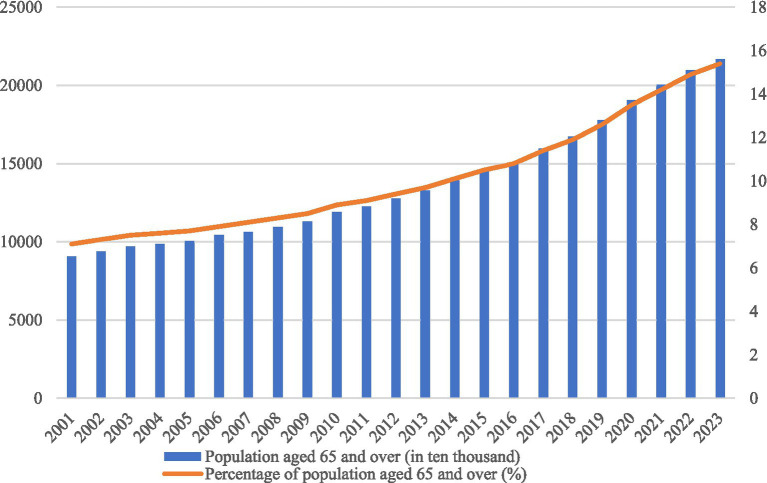
The development trend of China’s aging population.

Population aging represents one of the most profound demographic transformations of the twenty-first century, imposing complex challenges on social security, healthcare systems, and economic sustainability globally (1). However, for developing nations like China, this challenge is compounded by a unique “dual compression” effect: the country is experiencing rapid aging while simultaneously undergoing high-speed urbanization (2). Unlike developed nations that became wealthy before becoming old, China faces the structural dilemma of growing old before getting rich. By 2023, the proportion of the population aged 65 and above reached 15.4%, marking China’s entry into a moderately aged society. The accelerating trajectory of this demographic transition is illustrated in Figure 1, which visualizes the rapid accumulation of the older population over the past two decades. Yet, this national-level shift is not spatially uniform; rather, it exhibits strong regional heterogeneity. Coastal, economically developed regions and specific urban clusters show distinct aging patterns compared to inland and rural areas, driven by the complex migration of labor and the uneven distribution of public resources (3).

In this context, the determinants of aging are no longer limited to natural birth and death rates but are increasingly shaped by the quality of the living environment. As a core component of urban infrastructure, Urban Green Space (UGS) has emerged as a critical factor influencing residential satisfaction and population mobility. Traditional literature has extensively documented the micro level benefits of UGS, such as reducing air pollution, mitigating urban heat islands, and promoting physical and mental health among the older population (4, 5). However, these studies predominantly focus on the physiological health of individuals within a specific location. They often overlook a critical macro level mechanism: how the spatial distribution of green space acts as a magnet for resources and population, thereby reshaping the demographic structure across cities.

This oversight presents a significant research gap. In an era of high population mobility, UGS serves as a proxy for high quality life. Cities with abundant green resources tend to attract younger, skilled migrants seeking better living environments, which may dilute the local aging rate. Conversely, the “siphon effect” of these green hubs may accelerate aging in surrounding areas as they lose their working age population. Alternatively, high quality green cities may also attract retirees seeking “amenity migration,” thereby increasing local aging levels (6). Therefore, the relationship between UGS and aging is likely not a simple linear correlation but a complex process involving spatial spillovers and nonlinear thresholds.

Existing studies have largely failed to capture these spatial and nonlinear complexities. Most research employs ordinary least squares regression, which assumes independence among spatial units and ignores the spillover effects where green development in one city affects the demographic structure of its neighbors. Furthermore, few studies have examined the threshold effects of UGS. It remains unclear whether the impact of green space on aging changes structurally once green coverage exceeds a certain critical value.

To address the identified gaps in existing literature, this study provides three profound marginal contributions that enhance both the theoretical framework and the practical application of environmental demography. First, this research achieves a theoretical breakthrough by shifting the focus from localized, intra-city benefits to macro-scale spatial spillover effects. While prior studies predominantly examine the immediate health impacts of Urban Green Space (UGS) on local older populations, our study utilizes the Spatial Durbin Model to reveal how UGS functions as a regional amenity that mitigates aging pressures across administrative boundaries. This provides a new perspective on aging as a spatially clustered phenomenon, where environmental optimization in one city generates positive externalities for its neighbors. Second, we offer significant methodological depth by identifying the nonlinear dynamics of the UGS-aging relationship. Through threshold regression analysis, we demonstrate that the effectiveness of UGS is not constant but exhibits a critical tipping point. Specifically, we find that the positive impact on aging mitigation intensifies significantly once green space coverage surpasses a certain threshold, a discovery that offers a level of analytical precision missing from previous linear assessments. Third, the practical value of this study lies in its evidence-based framework for regional collaborative governance. By moving beyond isolated urban planning, our findings provide a scientific roadmap for the Healthy China 2030 strategy, emphasizing that balanced regional development and cross-boundary greening initiatives are essential to address the challenges of an aging society in an era of high population mobility.

## 2 Literature review and theoretical framework

### 2.1 Literature review

Urban green space (UGS), comprising parks, forests, wetlands, and other natural elements, is increasingly recognized as a critical determinant of public health, especially within the context of an aging population (7). As the proportion of older adults continues to rise globally, with China facing one of the most profound and rapid transitions, understanding how UGS contributes to physical, psychological, and social well-being has become a critical research frontier.

A major dimension of UGS lies in its capacity to regulate the ecological environment and reduce harmful exposures. Vegetation filters air pollutants through deposition and absorption, mitigates surface heat by altering microclimates, and moderates urban airflow dynamics, thereby alleviating health risks associated with respiratory diseases and heat-related illnesses (8). Empirical evidence demonstrates that urban greening improves air quality, decreases concentrations of particulate matter, and enhances overall life satisfaction (9). Furthermore, UGS functions as a natural cooling infrastructure, attenuating the urban heat island effect and lowering mortality risks during periods of extreme heat (10). Remote sensing-based spatial regression analyses demonstrate that increased vegetation coverage consistently mitigates heat stress exposure in cities worldwide (11). Collectively, these findings suggest that UGS serves as a vital environmental buffer, protecting older populations from pollution-induced morbidity and climate-induced vulnerabilities.

The contribution of UGS extends beyond environmental regulation to the promotion of physical activity, which is indispensable for healthy aging. Public green areas provide accessible and safe venues for low-intensity exercise that improve cardiovascular fitness, mobility, and overall physical resilience (12). Extensive epidemiological research further demonstrates that contact with greenery is associated with lower risks of chronic disease, reduced all-cause mortality, and improved immune function among the older population (13). In addition, by lowering concentrations of PM2.5, urban vegetation directly reduces the incidence of respiratory and cardiovascular complications (14). Park-based vegetation and tree belts within the urban core can effectively remove particulate matter, thereby producing measurable health gains for aging residents (47). These studies indicate that UGS is not only a leisure amenity but also an integral determinant of physical health outcomes among older adults.

Psychological and social dimensions represent another pathway through which UGS contributes to healthier aging. Contact with natural environments has been shown to alleviate stress, reduce depressive symptoms, and enhance cognitive restoration (15). Green buffers also attenuate noise pollution, which is a critical yet often overlooked determinant of neurological health, sleep quality, and emotional stability (16). Moreover, parks and green areas create venues for social interaction, which are essential for mitigating loneliness, reinforcing community cohesion, and sustaining intergenerational connections (17). During the COVID-19 pandemic, when social distancing disrupted conventional forms of interaction, UGS provided critical spaces for maintaining psychological well-being and physical activity (18). These findings emphasize the dual psychological and social mechanisms through which UGS enhances quality of life and prolongs functional independence in later life.

Nevertheless, the benefits of UGS are not equitably distributed across populations. Studies reveal that affluent neighborhoods are more likely to enjoy abundant and well-maintained green amenities, while marginalized or peripheral districts remain underserved (19). This inequitable distribution disproportionately affects older adults, who often face limited mobility, financial constraints, and accessibility barriers, thus preventing them from reaping the full health dividends of UGS (20). Consequently, rather than reducing inequalities, uneven access to UGS may inadvertently reinforce health disparities among aging populations. Addressing these inequities requires not only the quantitative expansion of UGS but also targeted planning strategies that prioritize vulnerable groups.

The salience of UGS is further amplified under the accelerating pressures of climate change. Older adults are particularly susceptible to extreme climatic events, including heat waves and heavy precipitation, due to diminished thermoregulatory capacity, a higher prevalence of chronic diseases, and reduced adaptive resilience (21). Green infrastructure provides critical adaptive functions by moderating thermal stress, reducing stormwater runoff, and supplying shaded refuges that decrease climate-related mortality (22). For instance, increased tree canopy coverage has been associated with reduced deaths during extreme heat episodes in densely populated urban neighborhoods. This underscores that UGS should not only be conceptualized as a health-promoting amenity but also as a strategic climate adaptation measure for safeguarding older populations.

The most recent academic discourse has shifted toward a more granular and dynamic understanding of how urban green infrastructure mitigates aging pressures. Emerging research utilizes high resolution satellite imagery and big data to confirm that the spatial configuration of green spaces, rather than just their total area, plays a decisive role in enhancing the mobility and life expectancy of the older population (23). Furthermore, smart green spaces can provide real time health monitoring and social prompts for aging populations (7). In the context of global climate change, the necessity of green spaces as critical urban cooling centers, which are essential for reducing heat related mortality among vulnerable older population (24). These contemporary findings underscore that the spatial and nonlinear effects of green space are increasingly influenced by technological integration and climate adaptation strategies, reinforcing the practical significance of our spatial econometric analysis.

Despite substantial progress, several gaps remain evident in the literature. Existing research is often confined to specific case studies, raising concerns about external validity. Methodologically, reliance on cross-sectional or survey-based data constrains the ability to establish causality and assess long-term impacts. Furthermore, while physical, psychological, and environmental benefits of UGS have been widely documented, there is insufficient attention to how these effects vary according to spatial accessibility, socioeconomic stratification, or differential exposure to climate extremes. These limitations highlight the necessity of adopting longitudinal datasets and advanced econometric models—such as spatial Durbin and threshold regression frameworks—to capture spillover effects and nonlinear dynamics more systematically.

### 2.2 Theoretical foundation and research hypotheses

**Figure 2:**
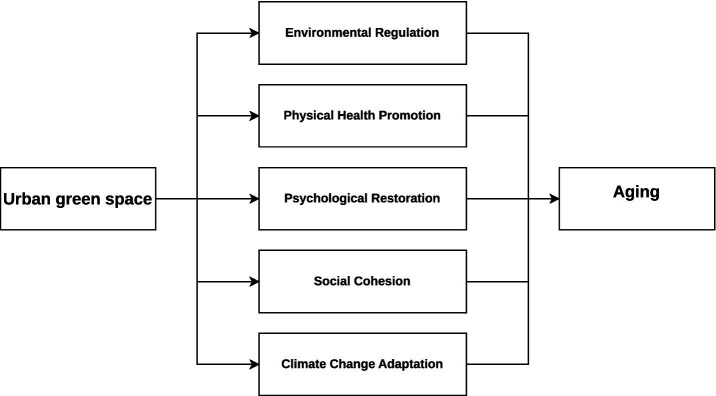
The logic of urban green space promoting aging.

As illustrated in Figure 2, urban green space influences aging outcomes through five major pathways—environmental regulation, physical health promotion, psychological restoration, social cohesion, and climate change adaptation—while also providing critical functions for climate adaptation. These pathways inform the following hypotheses, which guide the empirical analysis.

Urban green space plays a crucial role in promoting the physical health of older adults. By providing accessible environments for outdoor activities, green spaces reduce sedentary lifestyles, enhance cardiovascular fitness, and mitigate the prevalence of chronic diseases. At the same time, green vegetation improves local air quality by filtering pollutants and lowering exposure to harmful particles, which directly benefits the respiratory health of vulnerable populations. Environmental Restoration Theory suggests that natural environments provide essential resources for recovering from cognitive fatigue and physiological stress. In the context of aging, UGS functions as a “health stabilizer” by reducing the prevalence of chronic diseases and thermal stress among the older population. This improvement in physiological resilience directly influences demographic structures by reducing age-specific mortality and enhancing the local “age-in-place” capacity. Together, these mechanisms highlight the essential role of UGS in safeguarding physical health as populations age (25). Yet beyond its direct health benefits, the expansion of urban green space may also reshape demographic structures. Enhanced living environments and improved health resources in urban areas often attract younger cohorts, leading to rural out-migration and shifting population compositions. As a result, the relative share of older adults in the overall population may increase, reflecting the demographic consequences of urban green space development.

*Hypothesis 1: Urban green space significantly reduces the aging rate by optimizing the environmental determinants of health and increasing the survival quality of the older population.*

Beyond physical health, UGS also contributes substantially to the psychological and social well-being of the older population. Access to green environments helps reduce stress, alleviate depressive symptoms, and combat loneliness, which are key challenges in the context of population aging. In addition, UGS fosters opportunities for social interaction and community building, strengthening social cohesion and buffering the negative psychological effects of isolation among older adults. These findings suggest that UGS serves as a critical public health resource not only by promoting mental resilience but also by reinforcing social ties in aging communities (26). At the same time, the benefits of urban green space are not confined within administrative boundaries. Improvements in environmental quality, living conditions, and health resources in one city can influence migration flows and reshape demographic structures in neighboring regions. As urban centers enhance their attractiveness through expanded green spaces, surrounding areas may experience accelerated out-migration of younger populations, indirectly increasing the share of older adults. According to the Tiebout Model, residents “vote with their feet” to choose jurisdictions that offer their preferred mix of public goods. As UGS becomes a key regional amenity, it generates significant spatial spillovers. A city with high-quality green infrastructure not only benefits its own residents but also creates a regional “green magnet” effect. This attracts or retains older populations in broader urban clusters and facilitates cross-city health externalities through regional integrated networks. Therefore, the impact of UGS is not geographically encapsulated but extends to neighboring regions through population mobility and ecological connectivity. These interregional dynamic underscores the possibility that UGS exerts spatial spillover effects on demographic aging.

*Hypothesis 2: The impact of urban green space on China’s aging population exhibits a spatial spillover effect, that is, urban green space not only promotes the aging of the local population, but also promotes the aging of the population in surrounding areas.*

Furthermore, the role of UGS in the era of climate change cannot be overlooked. With rising global temperatures and the intensification of urban heat islands, older adults are disproportionately exposed to heat-related health risks (27). UGS can effectively mitigate these risks by regulating microclimates, reducing ambient temperatures, and supporting climate adaptation. However, the benefits of UGS may not increase linearly; instead, they may demonstrate threshold effects, where health improvements become more pronounced once green space availability surpasses a certain level. Moreover, spatial spillover effects imply that the benefits of UGS extend beyond local communities, influencing aging outcomes in neighboring areas. Taken together, these insights suggest that the demographic effects of UGS are likely to be nonlinear. At lower levels of green coverage, the capacity of UGS to influence population aging may be limited. Yet once a critical threshold is reached, the ecological and health benefits become strong enough to alter migration dynamics and amplify the concentration of older adults. This indicates that the relationship between UGS and demographic aging is characterized by a marginal increasing effect rather than a simple linear association.

*Hypothesis 3: The impact of urban green space on China’s aging population is nonlinear and shows a marginal increasing effect. That is, as urban green space increases, its impact on China’s aging population increases significantly after exceeding a certain threshold.*

Taken together, the reviewed literature underscores that UGS is a multidimensional determinant of aging, operating through environmental regulation, physical activity promotion, psychological restoration, social cohesion, and climate adaptation. At the same time, it reveals pressing gaps in methodological rigor and policy relevance, necessitating more systematic and spatially explicit research. By integrating these perspectives, this study provides a foundation for examining how urban green space can shape the trajectory of population aging in China, thereby informing both urban planning and public health policy.

## 3 Methodology

### 3.1 Model construction

Based on the theoretical analysis above, this study uses the Spatial Durbin Model (SDM) to explore the spatial spillover effects of urban green space on aging, and employs a threshold regression model to assess the nonlinear and phased nature of the impact of urban green space on aging. These two models form a robust analytical framework that not only examines the direct and indirect effects of urban green space on aging, but also examines how these effects vary across different stages of the urban green space development process.

#### 3.1.1 Baseline regression model

To empirically verify the hypothesis of a positive correlation between urban green space and aging proposed in the theoretical analysis, we constructed the following baseline regression model in Equation 1:


$$\text{agein}g_{\mathrm{it}}=\alpha_{0}+\alpha_{1}\mathrm{UG}S_{\mathrm{it}}+\alpha_{2}\text{control}s_{\mathrm{it}}+\mu_{i}+\varphi_{t}+\varepsilon_{\mathrm{it}}$$ (1)

where ageing_it_ represents the national aging level of spatial unit _i_ at time _t_, UGS_it_ represents the urban green space area of spatial unit _i_ at time _t_, and controls_it_ represents the control variable; α_0_ is a constant term, α_1_ and α_2_ are the coefficients of the core explanatory variable and the control variable, respectively; μ_i_ represents the region-specific fixed effect, which is used to control for unobservable time-invariant heterogeneity among regions; φ_t_ represents the time fixed effect, which is used to control for macroeconomic shocks and policy changes common to all regions; and ε_it_ is the random error term.

#### 3.1.2 Spatial Durbin model

Common spatial econometric models include the spatial lag model (SLM), the spatial error model (SEM), and the spatial Durbin model (SDM). The SDM is an extended and more flexible model that considers spatial lags in both the dependent and independent variables, effectively combining the structures of the SLM and SEM (28). This model can more comprehensively assess direct and indirect (spillover) effects across spatial units. This study uses the SDM to further examine the spatial spillover effects of urban green space on national aging. The model is detailed as follows in Equation 2:


$$\begin{array}{l} \text{agein}g_{\mathrm{it}}=\beta_{1}\sum_{i=1}^{n}W\times \text{agein}g_{\mathrm{it}}+\alpha_{1}\mathrm{UG}S_{\mathrm{it}}+\alpha_{2}\text{control}s_{\mathrm{it}}\\ +\beta_{2}\sum_{i=1}^{n}W\times \mathrm{UG}S_{\mathrm{it}}+\beta_{3}\sum_{i=1}^{n}W\times \text{control}s_{\mathrm{it}}+\mu_{i}+\varphi_{t}+\varepsilon_{\mathrm{it}} \end{array}$$ (2)

Where β_1_, β_2_, and β_3_ are spatial autoregressive coefficients, describing the influence of neighboring regions on local regions. W is a spatial weight matrix reflecting the spatial structure of interactions, which serves as the core mathematical structure for quantifying the spatial interactions among the 300 Chinese cities in our sample. This matrix is constructed using the queen contiguity principle, where a binary weight of one is assigned if two cities share a common boundary or vertex, and zero is assigned otherwise. To facilitate the interpretation of the spatial parameters and ensure the stability of the estimation, the matrix undergoes a row standardization process, effectively normalizing the spatial influence of neighbors. This structural configuration allows the model to capture the endogenous spatial dependencies of population aging and the exogenous spillover effects of urban green space across administrative boundaries. W × ageing_it_ represents the spatial lag of the national aging population, W × UGS_it_ represents the spatial lag of urban green space, and W × controls_it_ represents the spatial lag of the control variables. n is the number of spatial units. The definitions of the remaining variables are the same as in the baseline model. This study uses geographic and economic distance matrices to explain spatial relationships. The construction of these matrices follows the method proposed by Janatabadi and Ermagun (29).

#### 3.1.3 Threshold regression model

To examine the phased impact of urbanization on China’s aging population, this study further analyzes the threshold regression model proposed by Hansen (30), using urban green space as the threshold variable. This approach enables us to identify and quantify the potential nonlinear effects of urban green space development at different stages. The threshold regression model is as follows in Equation 3:


$$\begin{array}{l} \text{agein}g_{\mathrm{it}}=\lambda_{0}+\lambda_{1}\mathrm{UG}S_{\mathrm{it}}\times I(th_{\mathrm{it}}\leq \gamma_{1})+\lambda_{2}\mathrm{nUG}S_{\mathrm{it}}\\ \times I(\gamma_{1}\leq th_{\mathrm{it}}\leq \gamma_{2})+\cdots +\lambda_{n}\mathrm{nUG}S_{\mathrm{it}}\times I(\gamma_{n-1}\leq th_{\mathrm{it}}\leq \gamma_{n})\\ +\lambda_{n+1}\mathrm{UG}S_{\mathrm{it}}\times I(th_{\mathrm{it}}\geq \gamma_{n})+\alpha_{2}\text{control}s_{\mathrm{it}}+\varepsilon_{\mathrm{it}}\; \end{array}$$ (3)

where th_it_ is the threshold variable, and λ0 is the constant term; λ_1_, λ_2_, …, and λ_n + 1_ are the regression coefficients of the explanatory variables UGS_it_ at different threshold intervals; γ_1_, γ_2_, …, and γ_n_ are the threshold values to be estimated; I (·) is the indicator function that evaluates to 1 if the threshold variable satisfies the conditions in the parentheses, and 0 otherwise. This model helps to examine whether the impact of urban green space on China’s aging population increases, decreases, or undergoes a qualitative change at different stages of urban green space development.

### 3.2 Variable selection

In accordance with the research design, this study sets the explained variables, core explanatory variables and a series of control variables as follows:

1. Dependent variable: China’s aging level (aging). Population aging is usually measured by indicators such as the proportion of the older population, the old-age dependency ratio, and the median age of the older population (31). This study uses the proportion of the Chinese population aged 65 and above to the total population of China to measure China’s aging level. In addition, the median age of the older population is also used as a proxy variable for the robustness test of this paper.
2. Core independent variable: Urban green space (UGS). Here, urban green space is defined as a type of land within the urban planning area, with natural and artificial vegetation as the main body, used to improve the urban ecological environment, beautify the landscape, and provide leisure and recreation space. The statistical caliber of urban green space area is used (32).
3. Control variables. Although the direct drivers of China’s aging population include declining fertility and increasing life expectancy, its deeper roots lie in socioeconomic development and environmental changes (33, 48). To avoid estimation bias caused by omitted variables, based on data availability and previous research, the following 10 control variables are included in the model analysis (33–37). ① Economic development level (economy), measured by GDP per capita; ② Urbanization level (development), measured by the proportion of urban population in the total population; ③ Urban–rural relationship structure (relation), measured by the ratio of urban residents’ per capita disposable income to rural residents’ per capita disposable income; ④ Residents’ income (income), usually measured by residents’ per capita disposable income; ⑤ Residents’ consumption level (expend), usually measured by residents’ per capita expenditure; ⑥ Medical conditions (medical), measured by the number of beds in medical and health institutions per thousand people; ⑦ Education level (education), measured by the average years of education; ⑧ Traffic conditions (traffic), measured by road density, that is, the length of roads per unit area; ⑨ Rural revitalization policy (policy), measured by the rural revitalization development coefficient. To assess the impact of regional development policies, we employed the Rural Revitalization Development Coefficient. This coefficient is not a single indicator but a comprehensive index calculated using the entropy weight method, ensuring an objective weighting of various data. The construction of this index encompasses five key dimensions: industrial prosperity (agricultural output), ecological livability (waste management), cultural civilization (education facilities and cultural services), effective governance (administrative management), and life prosperity (farmers’ income). By integrating these indicators, this development coefficient provides a comprehensive measurement standard for the policy effectiveness of each region; ⑩ Air quality (environment), measured by the annual average PM2.5 concentration.

### 3.3 Data sources and processing

Although China’s provincial administrative boundaries have remained largely unchanged over the past few decades, the use of provincial data is limited by its coarse granularity, which hinders a detailed depiction of socioeconomic dynamics. While county-level data can provide finer details, frequent administrative adjustments, particularly redrawing, hinder cross-year comparability. In contrast, prefecture-level administrative divisions have undergone relatively few such changes in recent decades, making prefecture-level panel data more suitable for capturing the spatiotemporal variations in China’s socioeconomic development. Therefore, this study utilizes prefecture-level panel data from mainland China for 2001, 2013, 2018, and 2023 to explore the impact of urban green space on national aging. In the specific analysis, the four municipalities of Beijing, Tianjin, Shanghai, and Chongqing are treated as prefecture-level administrative divisions. Excluding regional units with missing or difficult-to-access data, this study includes 300 spatial units and 1,200 observations. The data used are from China Statistical Yearbook, China Health Statistics Yearbook, China Population and Employment Statistics Yearbook, China Labor Statistics Yearbook, National Economic and Social Development Statistics Bulletin, provincial and municipal Statistical Yearbooks, the National Bureau of Statistics, the database of the National Research Network, and the database of Guotai Junan. Table 1 lists all variable names and definitions used in the analysis.

**Table 1:** Variable definition.

Variable	Definition
China’s aging level (aging)	Proportion of rural population aged 65 and over (%)
Median age of the older population	The median age of the older population (%)
Urban green space (UGS)	Areas within urban environments covered by vegetation (km^2^)
Economic development level (economy)	GDP per capita (yuan/person)
Urbanization level (development)	Proportion of urban population to regional total population (%)
Urban–rural structure (relation)	Ratio of per capita disposable income of urban to rural residents
Residents’ income (income)	Per capita disposable income of residents (yuan/person)
Residents’ consumption level (expend)	per capita consumption per capita consumption
Medical conditions (medical)	Number of beds in health care institutions per 1,000 persons (bed/1000 persons)
Education level (education)	Average years of schooling (year)
Traffic conditions (traffic)	Length of highways per unit area (km/km^3^)
Rural revitalization policy (policy)	Rural revitalization development coefficient
Air quality (environment)	Annual average PM2.5 concentration (μg/m3)

## 4 Results and discussion

This section examines the spatiotemporal patterns of China’s aging population and urban green space, and uses panel data to examine the impact of urban green space on China’s aging population and its phased differences. Furthermore, this section conducts robustness tests to ensure the reliability of the empirical results and explores the spatial heterogeneity of the observed effects.

### 4.1 Spatial–temporal characteristics

**Figure 3:**
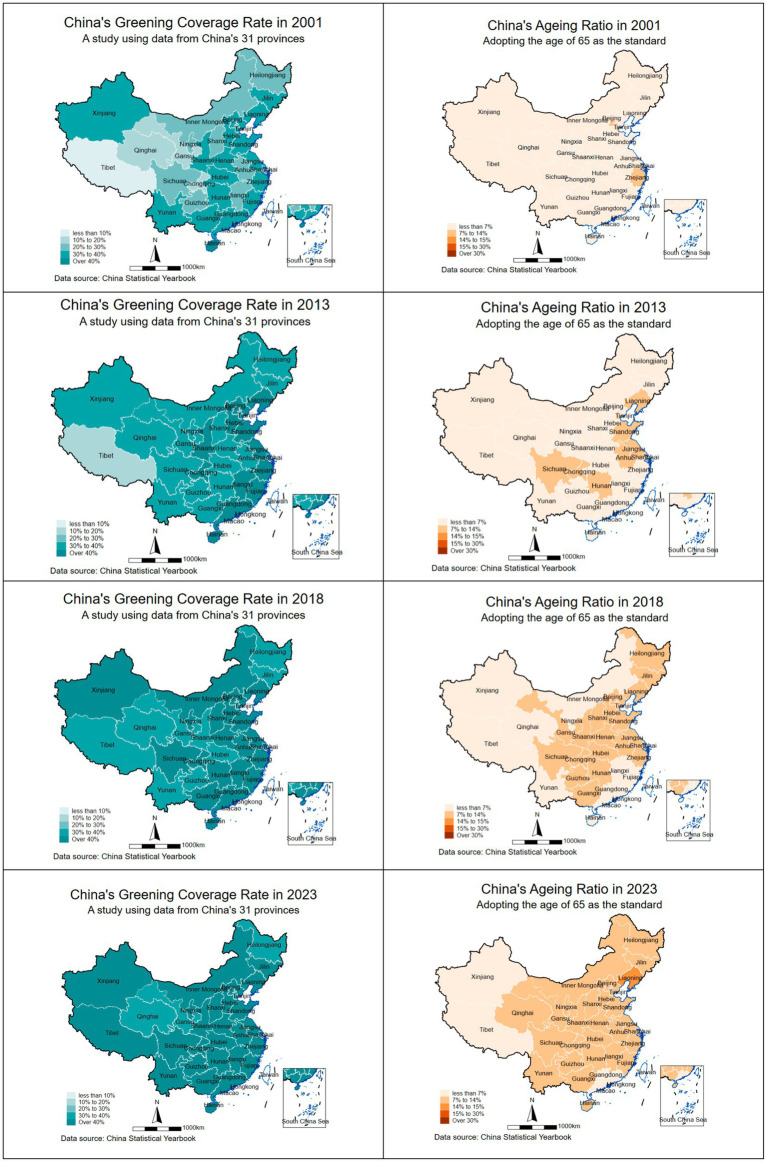
Key nodes of urban green space and aging in China’s development process.

The selection of 2001, 2013, 2018, and 2023 as research milestones stems from their role in clearly marking pivotal turning points in the interplay between China’s green space policies and its ageing process as shown in Figure 3. This trajectory commenced in 2001 with the ‘ecological compensation’ phase, prioritizing quantitative expansion. By 2013, as ‘ecological civilization’ ascended to national strategy, green space development shifted focus to quality and functionality, addressing public health demands amid accelerating ageing. By 2018, policy entered a new phase of ecological governance emphasizing both quantity and quality, confronting profound ageing challenges while highlighting green spaces’ health and social value. Finally, under the 2023 ‘dual carbon’ objectives, it advances towards a new ‘ecological welfare for the people’ phase that integrates ecological benefits with the needs of an ageing society. This represents a strategic evolution from merely increasing green coverage to constructing high-quality, age-friendly living spaces.

According to the 65-year-old standard, an ageing coefficient below 15% indicates a young population structure, 15–30% denotes a mature structure, and exceeding 30% classifies as an aged population structure. The United Nations (45, 46) defines a population as entering an ageing society when those aged 65 and over reach 7% of the total population, with 14% or above signifying a moderately aged society. Combining China’s and international statistical standards, the ageing coefficients are defined at 7, 14, 15, and 30%.

This paper also conducted a spatial autocorrelation analysis to examine the spatial clustering characteristics of China’s aging population. The results show that the Moran’s I index of rural aging, calculated based on the geographic distance matrix and the economic distance matrix, exceeded 0.5 in 2001, 2013, 2018, and 2023, and was significant at the 1% level. This indicates that the distribution of rural aging population levels in all three cross-sections exhibits significant positive spatial correlation. Notably, the Moran’s I index shows a sustained upward trend, indicating that the spatial clustering of China’s aging population has continued to strengthen during the observation period in Table 2. Similarly, the Moran’s I index of urbanization, calculated using both the geographic and economic distance matrices, remained above 0.17 in 2001, 2013, 2018, and 2023, with all values passing the 1% significance level.

**Table 2:** China’s aging level and urban green space’s Moran’s *I* index.

Year	Geographic distance matrix		Economic distance matrix	
	Ageing	UGS	Ageing	UGS
2001	0.5173***	0.2173***	0.5023***	0.3052***
2013	0.5898***	0.1898***	0.6021***	0.3188***
2018	0.5937***	0.1768***	0.6392***	0.3266***
2023	0.6139***	0.2035***	0.6656***	0.3444***

***p < 0.01, **p < 0.05, and *p < 0.1.

### 4.2 Spatial panel model results

#### 4.2.1 Model pre-validation

Spatial autocorrelation analysis results show that the Moran’s I index for China’s aging population and urban green space are both significantly positive, confirming the presence of spatial dependence and thus demonstrating the feasibility of spatial econometric models. This study first logarithmically transformed the four variables—economic development level, employment opportunities, rural residents’ income, and ecological environment—based on data attributes to reduce heteroskedasticity and enhance the robustness of the regression results. Subsequently, a multicollinearity diagnosis was performed to test for possible correlations between the variables, thereby avoiding issues such as unstable model estimation and unreliable hypothesis testing caused by collinearity. Variance inflation factor (VIF) results showed that the VIF values of all remaining variables were below 6 in Table 3. According to commonly used empirical rules (38), these results indicate that there is no significant multicollinearity among the remaining nine variables, making them suitable for subsequent regression analysis.

**Table 3:** Collinearity statistics.

Variable	VIF	1/VIF	VIF	1/VIF
UGS	2.07	0.4829	3.96	0.2525
Economy	2.05	0.4884	4.25	0.2353
Development	5.99	0.1669	5.86	0.1610
Relation	1.91	0.5255	2.79	0.3584
Income	2.32	0.4308	3.68	0.2717
Expend	3.85	0.2595	4.21	0.2375
Medical	4.25	0.2353	1.95	0.5128
Education	3.45	0.2899	2.51	0.3984
Traffic	5.52	0.1812	2.39	0.4184
Policy	2.36	0.4237	4.17	0.2398
Environment	5.54	0.1805	5.21	0.1919

We also conducted a series of diagnostic tests, including the Lagrange multiplier (LM) test, the likelihood ratio (LR) test, the Wald test, and the Hausman test, to determine the most appropriate spatial econometric model for the subsequent analysis. As shown in Table 4, the LM test results show that both the LM error and LM lag test statistics are significant at the 1% level, indicating that both the structural equation model (SEM) and the synthetic aperture radar (SAR) are suitable choices. The Hausman test supports the fixed-effects model, indicating that it is more appropriate than the random-effects model. Furthermore, both the LR test and the Wald test rejected the null hypothesis, supporting the use of the SDM model. Taken together, these results support the use of the SDM model with fixed effects to examine the spatial impact of urban green space on China’s aging population.

**Table 4:** Collinearity statistics.

Variable	Geographic distance matrix	Economic distance matrix
LM-Lag test	32.654***	31.254***
Robust LM-Lag test	11.453***	13.786***
LM-Error test	41.547***	40.754***
Robust LM-Error test	879.13***	863.463***
LR-Lag test	85.64***	46.25***
LR-Error test	45.21***	63.84***
Wald-Lag test	62.77***	38.59***
Wald-Error test	57.56***	69.54***
Hausman test	75.62***	86.53***

Results of the LM test, LR test, Wald test, and Hausman test under different weight matrices.

***p < 0.01, **p < 0.05, and *p < 0.1.

#### 4.2.2 Spatial econometric model results

This study first employs a two-way fixed-effects model to conduct the baseline regression. As reported in Table 5, the expansion of urban green space exerts a significantly positive influence on China’s aging population, and this effect remains robust regardless of whether control variables are incorporated, offering preliminary evidence in support of Hypothesis.1 Nonetheless, given that demographic and ecological processes do not occur in isolation, ignoring regional linkages may lead to biased estimations. In an open and interconnected regional context, urban green space is likely to generate spatial externalities that extend beyond administrative boundaries. To better capture these interdependencies, this study further adopts the Spatial Durbin Model (SDM), which allows for the identification of both direct effects within a region and spillover effects across regions. Specifically, spatial regression analyses are conducted using geographic and economic distance matrices to reflect different forms of regional proximity. The empirical estimates indicate that the coefficients of urban green space on population aging are 0.4637 and 0.2315 under the geographic and economic matrices, respectively, with both statistically significant at the 1% level. These findings provide robust evidence of positive spatial spillover effects, highlighting that the influence of urban green space is not confined to local areas but also radiates across regional boundaries. This reinforces the validity of Hypothesis 1 and underscores the necessity of incorporating spatial dependence when assessing the demographic implications of urban ecological resources.

**Table 5:** Benchmark regression results of the impact of urban green space on China’s aging population.

Variable	FE	FE	Geographic distance matrix	Economic distance matrix
UGS	0.6353***(0.3619)	0.5593***(2.6317)	0.4637***(0.3514)	0.2315**(3.3512)
Control variables	No	Yes	Yes	Yes
City fixed effect	Yes	Yes	Yes	Yes
Time fixed effect	Yes	Yes	Yes	Yes
N	1,200	1,200	1,200	1,200
*R* ^2^	0.7523	0.6367	0.5682	0.1357

*t*-statistics in parentheses; ***p < 0.01, **p < 0.05, and *p < 0.1; Yes and No indicate control and no control related variables or effects, respectively.

#### 4.2.3 Spatial effect decomposition

While the SDM estimation results confirm the existence of spatial interactions, the estimated coefficients of the explanatory variables cannot be directly interpreted as marginal effects, since they do not capture the partial derivatives of the dependent variable with respect to the independent variables. To address this limitation, the present study applies a partial differentiation approach, which allows for a decomposition of the total influence of urban green space on population aging into its direct and spillover components, as reported in Table 6.

**Table 6:** Spatial effects decomposition results.

Variable	Geographic distance matrix			Economic distance matrix		
	Direct effect	Spillover effect	Total effect	Direct effect	Spillover effect	Total effect
UGS	0.6328***(1.6349)	0.3625*(0.9351)	0.0325(0.6971)	0.4735***(2.3627)	0.1437**(1.8362)	0.4438**(3.5231)
Control variables	Yes			Yes		
City fixed effect	Yes			Yes		
Time fixed effect	Yes			Yes		
N	1,200			1,200		
R^2^	0.3681			0.5393		

*t*-statistics in parentheses; ***p < 0.01, **p < 0.05, and *p < 0.1; Yes and No indicate control and no control related variables or effects, respectively.

The results reveal that the direct effects of urban green space on aging are 0.0477 under the geographic distance matrix and 0.0446 under the economic distance matrix, both statistically significant. These findings indicate that the expansion of urban green space significantly accelerates the local aging process within a given region. In contrast, the spillover effects exhibit stronger variability. The coefficients of spatial spillover are 0.1437 for the geographic distance matrix and 0.4438 for the economic distance matrix, with statistical significance observed only in the latter. This pattern suggests that, beyond local impacts, urban green space exerts broader demographic externalities, particularly when regions are more closely connected through economic ties.

Moreover, the magnitude of the spillover effect in the economic space exceeds that of the direct effect, underscoring the intensification of demographic linkages across regions. This implies that urban ecological resources do not merely shape local demographic structures but also accelerate interregional aging dynamics through spatial interactions. Such evidence provides robust empirical validation of Hypothesis 2 and highlights the importance of considering both local and cross-regional effects when evaluating the demographic implications of urban ecological development.

### 4.3 Threshold regression model results

To further investigate the possibility of nonlinear dynamics between urban green space and China’s aging population, this study employs a threshold regression model to capture potential structural shifts in the relationship at different stages of development. Rather than relying on arbitrary interval classifications that may introduce estimation bias, we adopt the econometric framework developed by Hansen (30), which allows for the endogenous determination of threshold values. Specifically, tests for single, double, and triple thresholds of urban green space are conducted, and the statistical significance of the estimated thresholds is assessed through a bootstrap resampling procedure with 400 replications to ensure robustness.

The empirical results, presented in Table 7, indicate that the single- and double-threshold specification is statistically significant, while the triple-threshold models are not. This finding suggests that the relationship between urban green space and population aging is not uniform but instead varies across four distinct stages of urbanization. In other words, the demographic effects of urban green space intensify or diminish as the level of urbanization progresses, reflecting a nonlinear and stage-dependent mechanism. The presence of significant double-threshold effects provides compelling evidence that urban ecological expansion exerts heterogeneous influences on aging, thereby reinforcing the importance of accounting for structural heterogeneity when analyzing demographic transitions in the context of urbanization.

**Table 7:** Spatial effects decomposition results.

Threshold variable	Type	*F*-value	*p*-value	Critical value		
				10%	5%	1%
UGS	Single threshold	56.32	0.0000	21.956	18.302	14.628
	Double threshold	63.19	0.0008	18.531	16.531	15.364
	Triple threshold	26.58	0.1953	73.578	56.374	48.395

*t*-statistics in parentheses; ***p < 0.01, **p < 0.05, and *p < 0.1; Yes and No indicate control and no control related variables or effects, respectively.

### 4.4 Robustness test

To further validate the robustness and reliability of the empirical findings, this study implements three complementary strategies: variable substitution, variable shrinkage, and the exclusion of municipalities, as reported in Table 8.

**Table 8:** Robustness test.

Variable	Geographic distance matrix			Economic distance matrix		
	Variable substitution	Variable winsorization	Exclusion of municipalities	Variable substitution	Variable winsorization	Exclusion of municipalities
UGS	0.463*** (4.235)	0.539*** (5.631)	0.484*** (3.467)	0.632*** (4.849)	0.553*** (3.426)	0.467*** (0.863)
Control variables	Yes	Yes	Yes	Yes	Yes	Yes
City fixed effect	Yes	Yes	Yes	Yes	Yes	Yes
Time fixed effect	Yes	Yes	Yes	Yes	Yes	Yes
*N*	1,200	1,200	1,188	1,200	1,200	1,188
*R* ^2^	0.327	0.291	0.156	0.187	0.237	0.085

*t*-statistics in parentheses; ***p < 0.01, **p < 0.05, and *p < 0.1; Yes and No indicate control and no control related variables or effects, respectively.

First, variable substitution is conducted. Since population aging can be measured through multiple indicators, the dependent variable—China’s aging level—is replaced with the median age of the older population. The results demonstrate that the coefficient of urban green space remains significantly positive, thereby confirming the consistency of the baseline conclusions.

Second, variable shrinkage is applied to address the potential influence of extreme values. All variables are winsorized at the 1% bilateral quantile level, which reduces the sensitivity of the results to outliers. The re-estimated coefficients show that, under both geographic and economic spatial weight matrices, the impact of urban green space remains positive and statistically significant at the 1% level. This indicates that the main findings are robust to the presence of extreme observations.

Third, the exclusion of municipalities is undertaken. Given that municipalities differ substantially from ordinary prefecture-level cities in terms of economic structure, administrative role, and development advantages, their inclusion may bias the results. To mitigate this concern, municipalities are removed from the sample, and the estimations are repeated. The results continue to show that urban green space exerts a significant positive influence on China’s aging population, thereby strengthening the robustness and generalizability of the baseline findings.

These robustness checks corroborate the stability of the empirical analysis and reinforce confidence in the conclusion that urban green space contributes significantly to the dynamics of population aging in China.

### 4.5 Endogeneity test

In the spatial analysis of urban demographic structures, endogeneity often arises from the bidirectional relationship between environmental amenities and population distribution. While expansive green infrastructure may attract older residents to settle due to superior health benefits, the local aging level might also influence the prioritization of green space in urban planning, creating a reverse causality issue. To address this potential bias, we adopted the Two Stage Least Squares (2SLS) method. We utilized the urban green space lagged by one period (L.USG) as an instrumental variable for the current green space level. The theoretical justification is that historical green space development provides the ecological foundation for current aging patterns but is not influenced by contemporaneous demographic shocks. As shown in Table 9, the first stage results indicate a strong positive correlation between the instrument and the endogenous variable. In the second stage, the coefficient representing the impact of green space on population aging is positive and is significant at the 1 percent level. Based on a sample size of 1,200 observations, the Cragg Donald Wald F statistic of 28.12 exceeds the critical values for weak identification, confirming that our instrumental variable is both relevant and valid. This confirms that the positive role of green space in promoting the concentration of aging populations is robust even after controlling for endogeneity.

**Table 9:** Endogeneity test.

Variable	First stage	Second stage
L.USG	0.145*** (5.32)	–
USG	–	0.242*** (4.15)
Control variables	Yes	Yes
City fixed effect	Yes	Yes
Time fixed effect	Yes	Yes
*R* ^2^	0.685	0.762
*N*	1,200	1,200
Cragg Donald Wald *F*	28.12	

*t*-statistics in parentheses; ***p < 0.01, **p < 0.05, and *p < 0.1; Yes and No indicate control and no control related variables or effects, respectively.

### 4.6 Heterogeneity analysis

Given the vast geographical extent and uneven development levels in China, the impact of green space on population aging may exhibit significant regional heterogeneity. To explore these differences, we divided the 300 sample cities into three major regions: Eastern, Central, and Western China. As shown in Table 10, the effect of urban green space (USG) on the concentration of the older population is most pronounced in the Eastern region. This suggests that in developed Eastern cities, high quality ecological environments are a primary factor in residential choice for the aging population. Furthermore, the spatial spillover effect (W*USG) is significant in the Eastern region, indicating that green space expansion in one city significantly attracts older populations to neighboring areas within the same urban cluster. In contrast, the Central and Western regions show smaller coefficients respectively, and their spatial spillover effects are not statistically significant. This regional disparity confirms that the attractiveness of green infrastructure to the older population depends heavily on the local socioeconomic context and the overall maturity of the regional urban network. These findings provide a more nuanced understanding for policymakers to implement region specific urban greening strategies to address the challenges of an aging society.

**Table 10:** Heterogeneity analysis.

Variables	Eastern region	Central region	Western region
USG	0.285*** (5.21)	0.142** (2.15)	0.098* (1.82)
W*USG	0.115** (2.34)	0.045 (1.10)	0.012 (0.35)
Control Variables	Yes	Yes	Yes
City fixed effect	Yes	Yes	Yes
Time fixed effect	Yes	Yes	Yes
Observations	448	384	368
R-squared	0.821	0.712	0.685

*t*-statistics in parentheses; ***p < 0.01, **p < 0.05, and *p < 0.1; Yes and No indicate control and no control related variables or effects, respectively.

### 4.7 Discussion

#### 4.7.1 Rethinking the impact of urban green space on ageing in China

Since the beginning of the 21st century, the coverage of urban green space (UGS) in China has expanded steadily, while the problem of population ageing has intensified (39). This co-evolution is not coincidental, but rather reflects the deep interaction between ecological transformation and demographic restructuring. From the perspective of population health and mobility, UGS not only improves the environmental quality of cities and enhances their attractiveness to younger and economically active groups, but also reshapes fertility behavior, lifestyle preferences, and intergenerational relations by altering the broader social environment (35). These processes contribute to a ‘structural polarization’, whereby metropolitan areas concentrate younger and more productive populations, while rural and peripheral regions are left with disproportionately older residents.

Importantly, UGS is not simply an environmental amenity; it is also a mechanism of resource redistribution. Ecological assets, financial investment, and public services tend to cluster in urban cores, strengthening the advantages of metropolitan areas while weakening the demographic resilience of surrounding regions (25). In places where green infrastructure is scarce, the demographic consequences are often severe: declining populations, deteriorating health conditions, and a higher concentration of the older population and vulnerable groups (44). This process can be described as ‘selective sedimentation’, where green-rich cities attract mobile, productive cohorts, leaving behind the immobile, dependent, and older population (40). The outcome is an uneven demographic map marked by rapid and spatially unbalanced ageing.

The SDM results reinforce this interpretation by revealing significant spillover effects of UGS on ageing. Demographic shifts are not isolated; they unfold within networks of ecological and spatial interconnection. With the expansion of transport infrastructure, regional industry chains, and integrated environmental planning, green development in one area may displace demographic pressures onto others (41). For example, the establishment of green corridors and large urban parks can attract younger migrants from adjacent areas, accelerating ageing in less-developed regions. Similarly, rural land conversion for ecological projects may trigger population displacement, intensifying demographic decline in origin communities (18). Institutional asymmetries, particularly the entrenched urban–rural divide in public services and welfare systems, further exacerbate this mechanism. In this sense, areas with weaker green development often serve as ‘cost-bearing zones’ of urban ecological expansion, where demographic and economic burdens accumulate without sufficient compensation (15).

The threshold regression analysis provides further nuance, demonstrating that the impact of UGS on ageing is nonlinear and stage-dependent. At low levels of green coverage, the effect is weak and statistically insignificant. Once UGS surpasses a critical threshold, however, its influence intensifies rapidly. In the advanced stage, the effects extend beyond quantitative population shifts to involve qualitative changes in demographic structures. Peripheral areas experience shrinking demand as young cohorts relocate to green-rich cores, leaving behind a diminishing base of residents and weakened service provision. This dynamic produces a self-reinforcing cycle of ‘youth out-migration – service withdrawal – accelerated ageing’ (9, 42). If unchecked, such processes may lead to a ‘structural lock-in’, where ageing becomes entrenched and resistant to reversal, threatening the sustainability of both rural revitalization and urban development.

Taken together, these findings highlight that population ageing under the influence of UGS is the outcome of intertwined demographic, institutional, and spatial mechanisms. On the one hand, the attractiveness of green-rich urban centers draws in younger populations, while the older population, often with declining mobility and limited resources, remain in less-developed regions as family-based caregiving systems weaken. On the other hand, the fragmented social security system produces the widespread phenomenon of ‘working in the city, retiring in the countryside’, which exacerbates regional disparities in ageing (6). Additionally, ecological projects that require rural land conversion may deprive older residents of livelihood resources, further eroding the social and economic foundation of peripheral communities.

Therefore, ageing in the context of UGS expansion cannot be viewed merely as a demographic issue; it is the manifestation of systemic institutional gaps and spatial inequalities embedded in ecological development strategies. Addressing this challenge requires policy interventions that move beyond the sole expansion of urban green infrastructure. Instead, attention must be directed toward balanced ecological planning, equitable distribution of public services, and the integration of urban–rural systems to ensure that green development supports both environmental sustainability and demographic resilience.

#### 4.7.2 Policy implications

Population ageing in China should not be understood merely as a demographic inevitability but as a systemic outcome shaped by the interplay between urban green space (UGS) development, socioeconomic transformation, and institutional adaptation. The empirical findings of this study suggest that the expansion of UGS, while beneficial to health and environmental quality, can also trigger threshold effects. Once ecological advantages disproportionately concentrate in metropolitan cores, demographic imbalances may intensify, undermining traditional community-based support systems and aggravating regional ageing disparities. Addressing this paradox requires a proactive, multi-dimensional policy framework capable of integrating spatial, demographic, and ecological governance.

First, optimizing the spatial allocation of UGS is crucial to alleviating the demographic consequences of ‘selective migration’. Green-rich urban areas disproportionately attract younger and skilled populations, accelerating the depopulation of peripheral and rural regions and leaving them more vulnerable to ageing pressures. To counter this, green infrastructure should not be confined to metropolitan centers but expanded to township and county levels, ensuring equitable ecological welfare. Policy instruments such as targeted fiscal transfers, ecological compensation mechanisms, and rural land-use reform could incentivize local governments to embed green amenities into development strategies. Beyond infrastructure, fostering rural entrepreneurship and ecological industries can encourage return migration and sustain local livelihoods, thereby strengthening the endogenous attractiveness of less-developed regions and mitigating the centrifugal forces of metropolitan UGS concentration.

Second, metropolitan green strategies must incorporate interregional demographic linkages to establish a ‘regional ageing governance community’. This model emphasizes urban–rural reciprocity: metropolitan centers endowed with UGS radiate ecological, healthcare, and cultural resources, while surrounding areas provide complementary functions in food production, ecological preservation, and community-based older population care. To institutionalize this synergy, pension portability, healthcare coverage integration, and interjurisdictional fiscal sharing mechanisms are essential, ensuring that the migration of older populations does not exacerbate retirement burdens in sending regions. In this sense, UGS becomes not only an ecological asset but also a governance instrument to rebalance demographic pressures across regions.

Third, governance strategies must recognize the nonlinear nature of the UGS–ageing relationship and adopt a phased, differentiated approach. In regions with limited UGS, priorities should focus on establishing basic ecological infrastructure, affordable healthcare, and community-level support systems to stabilize populations. Intermediate-level UGS regions should emphasize structural reforms, such as integrating ecological industry with intergenerational care, to prevent population hollowing during transitional phases. In high-UGS regions, the challenge shifts toward redistributing ecological dividends: mechanisms should channel financial surpluses and ecological capacities from urban cores to peripheral areas, reinforcing interregional equity and avoiding demographic overconcentration. By embedding ageing governance into ecological transitions, this phased strategy can transform UGS development from a centrifugal force of migration into a centripetal driver of demographic balance.

Finally, the governance of ageing in the context of UGS expansion requires a paradigmatic shift toward a holistic, life-course-oriented framework. Ageing should be seen not only as a late-life challenge but as a cumulative process shaped by spatial exposure to ecological environments, access to healthcare, and intergenerational mobility. This perspective calls for integrated reforms across housing, land, health, and social security systems, redefining urban–rural relations as a shared ecological responsibility. Only through institutional innovation, spatial coordination, and ecological redistribution can China mitigate the structural risks of population ageing while aligning with broader objectives of rural revitalization, common prosperity, and sustainable development.

#### 4.7.3 Research limitations and future prospects

This study confirms the multi-level and multi-dimensional nature of the impact of urban green space on aging and reveals the complex spatial diffusion and nonlinear nature of this relationship. However, this study still has several limitations, which suggest directions for future research. First, the use of prefecture-level panel data may mask important heterogeneity at the micro level. Future research could incorporate more granular household or community-level data to better capture local population-environment interactions. Furthermore, expanding temporal coverage and increasing data frequency would help reveal more nuanced and evolving mechanisms.

Second, while the model includes a series of control variables covering social, economic, and environmental dimensions to mitigate endogeneity, it does not account for the potential influence of soft factors such as cultural attitudes, environmental awareness, or policy implementation capacity. Future research could apply more sophisticated quantitative and qualitative methods to explore how these contextual variables moderate the relationship between urban green space and population aging.

Third, while China’s experience in urban green space and aging governance provides valuable insights, significant institutional and developmental differences exist across countries (43). Therefore, cross-national comparative studies could be conducted to examine the universality and specificity of the relationship between urban green space and aging across different institutional contexts.

Overall, future research should strive to develop a more nuanced, interactive, and comparable framework for understanding population aging in the context of ecological and spatial transformations. This would not only deepen academic insights but also provide richer empirical guidance for policymaking in demographic management and environmental planning in both developed and developing economies.

## 5 Conclusion

This study employs prefecture-level panel data from China (2001–2023) and applies the Spatial Durbin Model (SDM) together with threshold regression to examine the impact of urban green space (UGS) on population ageing. The findings reveal the complex spatially nonlinear dynamics of demographic change under expanding UGS. The main conclusions are as follows:

1. UGS and ageing exhibit pronounced spatial heterogeneity and clustering. Between 2001 and 2023, rural ageing intensified significantly, with Moran’s I confirming strong positive spatial autocorrelation, characterized primarily by high–high and low–low clustering. Over the same period, UGS coverage expanded steadily.
2. UGS significantly accelerates ageing and generates strong spatial spillover effects. By inducing ‘selective migration’, UGS promotes the outflow of working-age populations and transfers ageing pressures across regions through transport, institutional, and economic linkages. SDM results indicate that the spillover effect is larger than the direct effect, underscoring the critical role of interregional connectivity in shaping demographic dynamics.
3. The relationship between UGS and ageing is nonlinear, with increasing marginal effects. Threshold regression results show that once ageing surpasses a critical level, a self-reinforcing ‘ageing trap’ may emerge, driven by population decline and contraction of service industries.

Overall, this study advances theoretical and empirical understanding of the UGS–ageing nexus by moving beyond linear assumptions and uncovering the dual mechanisms of spatial diffusion and nonlinear amplification. The findings provide a policy framework for targeted interventions, including optimizing UGS allocation, enhancing regional coordination, and adopting phased and differentiated governance to mitigate ageing risks and alleviate labor force imbalances.

## Data Availability

The data analyzed in this study is subject to the following licenses/restrictions: if data is required, please contact the corresponding author. Requests to access these datasets should be directed to Dongmei Zhang, erinzxn@163.com.

## References

[1] Maravilla NMAT Tan MJT. On demographic transformation: why we need to think beyond silos. Front Aging. (2025) 6:1659284. doi: 10.3389/fragi.2025.1659284, 41602163 PMC12832767

[2] Zhao Y Li J. Opportunities and challenges of integrating artificial intelligence in China's elderly care services. Sci Rep. (2024) 14:9254. doi: 10.1038/s41598-024-60067-w, 38649405 PMC11035568

[3] Zhang L Ren H Li C. Study on the development characteristics and spatial and temporal patterns of population ageing in 31 central cities in China. Front Public Health. (2024) 12:1341455. doi: 10.3389/fpubh.2024.1341455, 38699420 PMC11063271

[4] Thompson R Smith RB Karim YB Shen C Drummond K Teng C . Noise pollution and human cognition: an updated systematic review and meta-analysis of recent evidence. Environ Int. (2022) 158:106905. doi: 10.1016/j.envint.2021.106905, 34649047

[5] Tian W Cao K Kwan MP Chiu MYL Chen H. How does urbanization affect the cognitive function among older adults: a geospatial analysis in China. Health Place. (2024) 88:103259. doi: 10.1016/j.healthplace.2024.103259 38776750

[6] Tabrizi N. Lak A. Moussavi A, S. M. R. (2023). Green space and the health of the older adult during pandemics: a narrative review on the experience of COVID-19. Front Public Health, 11:1218091. 37601191 10.3389/fpubh.2023.1218091 PMC10433209

[7] Ali MJ Rahaman M Hossain SI. Urban green spaces for elderly human health: a planning model for healthy city living. Land Use Policy. (2022) 114:105970. doi: 10.1016/j.landusepol.2021.105970

[8] Zhao T Heinrich J Brauer M Fulman N Idrose NS Baumbach C . Urban greenspace under a changing climate: benefit or harm for allergies and respiratory health? Environ Epidemiol. (2025) 9:e372 39957764 10.1097/EE9.0000000000000372 PMC11826049

[9] Wu L Chen C. Does pattern matter? Exploring the pathways and effects of urban green space on promoting life satisfaction through reducing air pollution. Urban For Urban Green. (2023) 82:127890. doi: 10.1016/j.ufug.2023.127890

[10] Pereira C Flores-Colen I Mendes MP. Guidelines to reduce the effects of urban heat in a changing climate: green infrastructures and design measures. Hoboken, NJ: John Wiley & Sons. Sustain Dev. (2024) 32:57–83.

[11] Massaro E Schifanella R Piccardo M Caporaso L Taubenböck H Cescatti A . Spatially-optimized urban greening for reduction of population exposure to land surface temperature extremes. Nat Commun. (2023) 14:2903. doi: 10.1038/s41467-023-38596-1, 37217522 PMC10203342

[12] Li C Lee CW Tsang KT. Outdoor walking better? Environmental elements of cardiorespiratory fitness training trails. Front Sports Active Living. Hoboken, NJ: John Wiley & Sons. (2023) 4:1036777. doi: 10.3389/fspor.2022.1036777, 36699982 PMC9870626

[13] Roscoe C Mackay C Gulliver J Hodgson S Cai Y Vineis P . Associations of private residential gardens versus other greenspace types with cardiovascular and respiratory disease mortality: observational evidence from UK biobank. Amsterdam: Elsevier. Environ Int. (2022) 167:107427 35905597 10.1016/j.envint.2022.107427

[14] Farley KL Owen D Fitch A Fletcher D Scheffler J Jones L. A tale of two cities: contrasting equity of greenspace benefits in relation to PM2. 5 exposure. BMC Public Health. (2025) 25:3449. doi: 10.1186/s12889-025-23813-x, 41068655 PMC12512593

[15] Li H Dong W Wang Z Chen N Wu J Wang G . Effect of a virtual reality-based restorative environment on the emotional and cognitive recovery of individuals with mild-to-moderate anxiety and depression. Int J Environ Res Public Health. (2021) 18:9053. doi: 10.3390/ijerph18179053, 34501643 PMC8430968

[16] Chu S Xu W Zhang D Lin J Liu J Liu S . Urban blue-green spaces and tranquility: a comprehensive review of noise reduction and sensory perception integration. J Asian Archit Build Eng. Abingdon: Taylor & Francis. (2025):1–22. doi: 10.1080/13467581.2024.2443048

[17] Shabani A Taheri S Istrate AL Sharifi A. Sense of belonging and attachment in urban green spaces: a qualitative study of older adults in Isfahan, Iran. Urban Stud. London: SAGE Publications. (2026):00420980251396546

[18] Naghibi M Farrokhi A Faizi M. Small urban green spaces: insights into perception, preference, and psychological well-being in a densely populated areas of Tehran, Iran. Environ Health Insights. (2024) 18:11786302241248314. doi: 10.1177/11786302241248314, 38756542 PMC11097736

[19] Kotsila P Anguelovski I García-Lamarca M Sekulova F. Injustice in urban sustainability: ten core drivers. London: Routledge: Taylor & Francis (2023). p. 170.

[20] Bressane A dos Santos Galvão AL Loureiro AIS Ferreira MEG Monstans MC de Castro Meiros LC. Valuing urban green spaces for enhanced public health and sustainability: a study on public willingness-to-pay in an emerging economy. Urban For Urban Green. Munich: Elsevier GmbH. (2024) 98:128386

[21] Vecellio DJ Vanos JK. Aligning thermal physiology and biometeorological research for heat adaptation and resilience in a changing climate. J Appl Physiol. (2024) 136:1322–8. doi: 10.1152/japplphysiol.00098.2024, 38385187 PMC11365541

[22] Pandey B Ghosh A. Urban ecosystem services and climate change: a dynamic interplay. Front Sustain Cities. (2023) 5:1281430. doi: 10.3389/frsc.2023.1281430

[23] Wang Z Gao L Song P. Impact of green infrastructure in smart older adult care communities on the health of the older adult and the exploration of optimization paths. Front Public Health. (2025) 13:1601102. doi: 10.3389/fpubh.2025.1601102, 40552226 PMC12183187

[24] Vasconcelos L Langemeyer J Cole HVS Baró F. Nature-based climate shelters? Exploring urban green spaces as cooling solutions for older adults in a warming city. Urban For Urban Green. (2024) 98:128408. doi: 10.1016/j.ufug.2024.128408

[25] Veen EJ Ekkel ED Hansma MR de Vrieze AG. Designing urban green space (UGS) to enhance health: a methodology. Int J Environ Res Public Health. Basel: MDPI. (2020) 17:5205 32708503 10.3390/ijerph17145205 PMC7400363

[26] Hossen MS Pauzi HBM Salleh SFB. Enhancing elderly well-being through age-friendly community, social engagement and social support. Am J Sci Educ Res. Selangor: Asiatic Scientific Publishers. (2023) 135, 1–10.

[27] Leal Filho W Icaza LE Neht A Klavins M Morgan EA. Coping with the impacts of urban heat islands. A literature based study on understanding urban heat vulnerability and the need for resilience in cities in a global climate change context. J Clean Prod. (2018) 171:1140–9. doi: 10.1016/j.jclepro.2017.10.086

[28] LeSage J Pace RK. Introduction to Spatial Econometrics. In: eds. D. B. Owen, J. N. Srivastava, and N. R. Draper, Series. Boca Raton: Chapman and Hall/CRC (2009).

[29] Janatabadi F Ermagun A. Access weight matrix: a place and mobility infused spatial weight matrix. Geogr Anal. (2024) 56:746–67. doi: 10.1111/gean.12395

[30] Hansen BE. Threshold effects in non-dynamic panels: estimation, testing, and inference. J Econom. Amsterdam: Elsevier. (1999) 93:345–68.

[31] Chang AY Skirbekk VF Tyrovolas S Kassebaum NJ Dieleman JL. Measuring population ageing: an analysis of the global burden of disease study 2017. Lancet Public Health. London: Elsevier. (2019) 4:e159–67. 30851869 10.1016/S2468-2667(19)30019-2 PMC6472541

[32] la De Barrera F Reyes-Paecke S Banzhaf E. Indicators for green spaces in contrasting urban settings. Ecol Indic. Amsterdam: Elsevier. (2016) 62:212–9.

[33] Burholt V Dobbs C. Research on rural ageing: where have we got to and where are we going in Europe? J Rural Stud. (2012) 28:432–46. doi: 10.1016/j.jrurstud.2012.01.009

[34] Borzoiepour S Alizadeh G Jafary H Zarnaq RK. Identify affecting factors on total fertility rate: a systematic review. Health Scope. The Hague: Brieflands. (2024) 13:e139351

[35] Cohen SA Greaney ML. Aging in rural communities. Curr Epidemiol Rep. New York: Springer. (2023) 10:1–16. 36404874 10.1007/s40471-022-00313-9 PMC9644394

[36] Ding W Zhang Y Zhang L Wang Z Yu J Ji H. Successful aging and environmental factors in older individuals in urban and rural areas: a cross-sectional study. Arch Gerontol Geriatr. Shannon: Elsevier. (2020) 91:104229 32871304 10.1016/j.archger.2020.104229

[37] Xu X Wang Y. Measurement of China's rural revitalization level, decomposition of regional differences and dynamic evolution. J Quantit Technic Econ. Beijing: Science Press. (2022). 39:64–83.

[38] Dormann CF Elith J Bacher S Buchmann C Carl G Carr’e G . Collinearity: a review of methods to deal with it and a simulation study evaluating their performance. Ecography. Hoboken: John Wiley & Sons. (2013) 36:27–46.

[39] Cai F Giles J O’Keefe P Wang D. (Eds.). The Elderly and old age Support in rural China. Washington, DC: World Bank Publications (2012).

[40] Karottki DG Spilak M Frederiksen M Gunnarsen L Brauner EV Kolarik B . An indoor air filtration study in homes of elderly: cardiovascular and respiratory effects of exposure to particulate matter. Environ Health. London: BioMed Central. (2013) 12:116. 24373585 10.1186/1476-069X-12-116 PMC3893545

[41] Dzhambov AM Dimitrova DD. Urban green spaces′ effectiveness as a psychological buffer for the negative health impact of noise pollution: a systematic review. Noise Health. (2014) 16:157–65. doi: 10.4103/1463-1741.134916, 24953881

[42] Aronson MF Lepczyk CA Evans KL Goddard MA Lerman SB MacIvor JS . Biodiversity in the city: key challenges for urban green space management. Front Ecol Environ. (2017) 15:189–96. doi: 10.1002/fee.1480

[43] United Nations Economic and Social Commission for Asia and the Pacific. (2024). Asia-Pacific report on population ageing 2022: Trends, policies and good practices regarding older persons and population ageing. Bangkok: United Nations.

[44] Fang J Liu Y An Y Zhou K. The macroeconomic impact of demographic shifts: aging populations and their socioeconomic consequences. Law Econ. (2023) 2:37–43. doi: 10.56397/le.2023.11.05

[45] United Nations (UN). World social Report 2023: Leaving no one behind in an Ageing World. Department of Economic and Social Affairs. New York: United Nations Publications. (2023).

[46] United Nations (UN). (2024). World Population Prospects 2024: Summary of Results. Department of Economic and Social Affairs. New York: United Nations Publications.

[47] Zhang X Wang C Guo J Zhu Z Xi Z Li X . Factors affecting dust retention in urban parks across site and vegetation community scales. Forests. (2024) 15:2136. doi: 10.3390/f15122136

[48] Glasgow N Brown D. L. Rural ageing in the United States: Trends and contexts. J. Rural Stud. (2012) 28:422–431.

